# The Efficacy of Fish as an Early Complementary Food on the Linear Growth of Infants Aged 6–7 Months: A Randomised Controlled Trial

**DOI:** 10.3390/nu14112191

**Published:** 2022-05-25

**Authors:** Given Chipili, Averalda Van Graan, Carl J. Lombard, Evette Van Niekerk

**Affiliations:** 1 Department of Nutritional Sciences, Mukuba University, Itimpi, Off Chingola Road, P.O. Box 20382, Kitwe 50100, Zambia; 2 Division of Human Nutrition, Faculty of Medicine and Health Sciences, Stellenbosch University, Francie Van Zijl Drive, Tygerberg 7505, Cape Town P.O. Box 19063, South Africa; averalda.vangraan2@mrc.ac.za (A.V.G.); evettev@sun.ac.za (E.V.N.); 3 South African Food Data System (SAFOODS), Biostatistics Unit, South African Medical Research Council, Francie Van Zijl Drive Parow Valley, Tygerberg 7505, Cape Town P.O. Box 19070, South Africa; 4 Division of Biostatistics, Faculty of Medicine and Health Sciences, Stellenbosch University, Francie Van Zijl Drive, Tygerberg 7505, Cape Town P.O. Box 19063, South Africa; cjlombard@sun.ac.za

**Keywords:** fish, complementary feeding, infants, growth, Zambia

## Abstract

Fish is a good animal-source protein for growth and development. The main objective of the study was to assess the efficacy of fish during the early complementary feeding period on infants’ linear growth in the Samfya district of the Luapula Province of Zambia in 6 months randomised controlled trial. The study was conducted from April 2019 to January 2020. Infants aged 6–7 months (*N* = 238) were assigned to either the intervention (treatment) group or control (placebo) group to receive fish powder or sorghum powder, respectively. Participants were followed on a weekly basis to distribute the powder and record compliance/usage and any morbidities. Anthropometric measurements were taken monthly. A linear mixed-effects model showed that fish powder improved linear growth among infants over all the 6 months of the intervention period. The fish powder increased length-for-age z scores by 1.26 (95% CI: 0.94–1.57) and weight-for-age z score by 0.95 (95% CI 0.6–1.23). The addition of fish powder to the infant’s usual food during the early complementary feeding improves the infant’s linear growth outcome.

## 1. Introduction

Malnutrition is one of the major causes of death in children below the age of five [1,2]. Stunting is a form of malnutrition that has affected over 151 million (22.2%) children under the age of five globally [3]. A child is considered to be stunted if their height for age is below the standard deviation (SD) of minus two of the World Health Organisation (WHO) median growth reference [4]. Stunting has both short-term and long-term consequences on children. Short-term consequences include poor cognitive, motor, and language development and a weakened immune system [5,6], while long-term consequences of stunting include poor school performance, short stature in adulthood, lost productivity, higher risks of diabetes, and cancer in adulthood [7,8,9]. The majority of stunted children are found in Sub-Saharan Africa, with 39% being [8] stunted, 15% underweight, and 10% wasted [10,11]. WHO has set a target to reduce stunting levels to 40% by 2025 [12,13].

In Zambia 35% of the children under the age of five are stunted, 12% are underweight, and 3% are wasted. Luapula Province, where the current study was conducted in a district called Samfya, recorded a rise in the levels of stunting from 43% in 2013 to 44.9% in 2018 [14]. In 2015 the Civil Society for scaling-up nutrition reported stunting levels of 49% in the Samfya district, making it one of the districts with the highest prevalence of stunting in the country [15].

Complimentary feeding plays a very important role in child development and the reduction of stunting levels. Complementary feeding is the introduction of solid/semisolid food to breastfed infants at the age 6 months when breast milk alone becomes insufficient to meet the nutritional requirements of infants [16,17]. Complementary diets for infants and young children in most developing countries, including Zambia, are plant-based and of low nutrient and energy density [18]. Studies have shown that children whose diets include animal-source protein foods are more likely to grow taller compared to the ones that are fed plant source protein [18]. Animal source food such as meat, poultry, and milk products are of great benefit in early complementary feeding but may be unaffordable to low-income groups [19]. However, utilizing locally available animal-source protein foods may overcome the barrier of food costs [20,21,22,23].

In the Samfya district, one such food source is small fish called chisense. Chisense is a small freshwater fish found in lake Bangweulu of Samfya district, which is usually sun-dried and consumed by the local people throughout the year. It is rich in zinc, protein essential amino acids, and calcium because it is eaten with bones [23]. Even though chisense is a well-known fish eaten by local people in the Samfya district, there has to date been no research conducted on its efficacy in improving growth when introduced in the early complementary feeding period.

## 2. Materials and Methods

### 2.1. Study Area

The study was conducted at Shikamushile Rural Health Centre (RHC), Samfya district of Luapula Province, Zambia, from April 2019 to January 2020. The centre serves 77 villages that are divided into eight (8) Zones with a population of 18,300, of which 3660 (20%) are children below the age of five years. The occupants are low-income families who depend on subsistence fishing and farming for their livelihood. There is abundance of fish in the community throughout the year as most of the fish caught is usually preserved through smocking and sun drying. Children in this community are usually given plain cassava or maize porridge as the main complementary food.

### 2.2. Participants and Study Design

A 6 months, single-blind randomised controlled trial (RCT) was conducted. The trial had two arms, with the intervention group receiving fish powder while the control was given a placebo in the form of sorghum powder. Data were collected at Shikamushile RHC and at the participant’s households. Infants aged 6–7 months that attended Shikamushile RHC were eligible to partake in the study. Exclusion criteria of the study were: (i) congenital diseases/disorders that affect growth; (ii) prematurity; (iii) HIV exposure or infection. Infants were exited from the study if: (i) they had a positive skin prick test (SPT) for fish allergy [24], (ii) mothers decided not to let their infant continue with their study; (iii) non-compliance to the intervention for more than two consecutive weeks were found and (iv) infants were severely malnourished and required hospitalisation. If there were two infants from the same household or, in the case of twins, they were allocated to the same group to avoid cross-contamination within groups. However, each child had a unique participant code and was assessed individually.

### 2.3. Sample Size Determination

The sample size calculation was based on an expected difference of 0.45 in length for age z-score (LAZ) between the intervention and control group over a period of 6 months at 0.05 level of significance, with 80% power using a 2-sample *t*-test. The sample size calculated was 95 infants per study arm. In order to compensate for an expected attrition rate of 20%, 119 infants were enrolled in each study arm. Therefore, the total sample was 238 infants.

**Figure 1:**
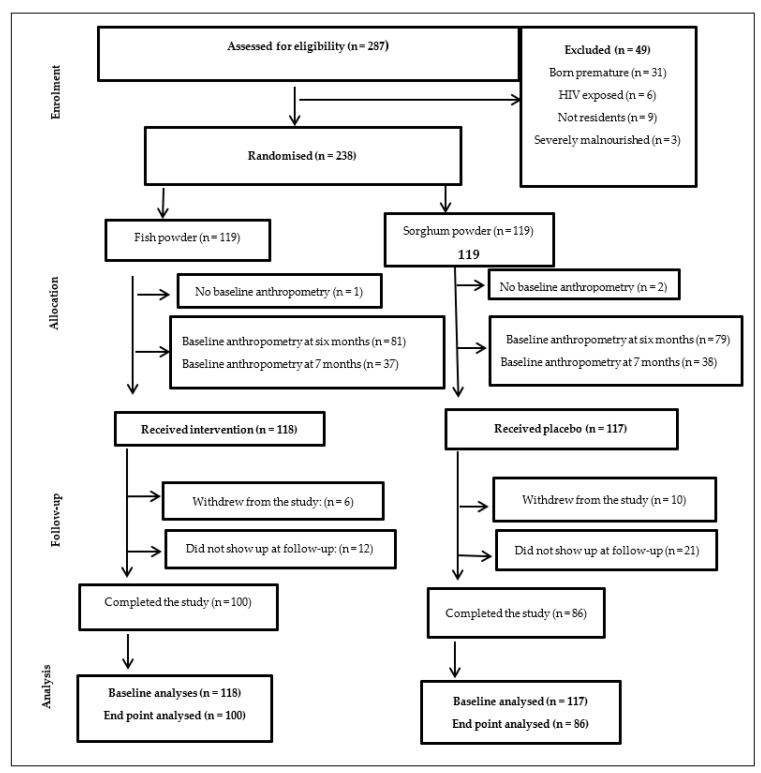
Flow of the study participants.

An independent statistician who was not part of the study prepared the randomization schedule. All eligible infants were randomly assigned to either the intervention (fish powder) or control (sorghum powder) group at 6–7 months. A 1:1 ratio using a random number generator and block randomization in Microsoft Excel was used to allocate the infants to two groups of equal size. The schedule was stratified by the gender of infants (boy or girl) to enable gender balance in both groups. Allocation of concealment was performed using a central randomisation service through a computer algorithm to protect the randomisation schedule. Two hundred and thirty-eight (238) infants were assigned randomly to either intervention (*n* = 119) or control group (*n* = 119) (Figure 1).

### 2.4. Participant Recruitment

The research team made announcements of the study at Shikamushile RHC during monthly under-five clinic visits and advertised through posters. All mothers with infants below six months were invited to participate in the study. Registration of participants was conducted by research assistant (part of the research team) at the RHC. Once the infants reached the age of six months, they were enrolled in the study if they met the inclusion criteria and the mothers provided written informed consent. Participants were recruited in four groups over four months. We enrolled 78 infants in the first month, 47, 51, and 62 in the second, third, and fourth months, respectively, to meet a sample of 238 infants. Before enrolment, mothers were invited to an information session where the study’s nature and eligibility criteria were explained. There were four information sessions (one per month) to cater to the four groups of mother–infant pairs in the study. The chief investigator also explained the content of the consent form. Those who signed the consent forms were given a copy and were included in the study.

Initially, we planned to enrol 40 mother–infant pairs per month for a period of six months to meet our sample size of 238. However, we received overwhelming response during the first month, and we enrolled 78 mother/infant pairs. Control measures and quality assurance of the first 78 infants showed inconsistencies which led to the extension of the intervention for the first group of an additional month using the 7-month measurements as a baseline. (Table 1).

### 2.5. Preparation of the Fish and Sorghum Powders

After purchase of dried fish/sorghum from the local market in Samfya district, the principal investigator, with the help of Makluba University lab assistants (not part of the research team), prepared the fish and sorghum powder in the Food Service Lab, Department of Nutritional Science, Makluba University in Kitwe, Zambia. Standard food safety and hygienic measures were ensured by cleaning and disinfecting the lab. The fish/sorghum was cleaned and placed in the oven at 280 °C for 10 min (fish) and 15 min (sorghum). The lab assistants roasted the dried fish/sorghum, cooled, and then ground it into powder using two separate portable local grinding mills for the two products.

The principal investigator (PI), with the help of Mukuba University lab assistants, prepared the sorghum product to ensure similarity in physical appearance and aroma with the *chisense* (fish) product. During grinding of sorghum powder, *chisense* fish (3 g/100 g of sorghum) were added to the sorghum to provide a similar fish aroma as the fish product. Therefore, only 0.2 g of fish was added per 7 g of sorghum powder, and the change in the nutritive value of the sorghum was negligible. The fish powder (12 g) and sorghum powder (7 g) were then pre-packed in small plastic sachets.

The participants and the field workers (research assistants and health workers) were blinded, while the (PI) was not. We used single blind to avoid exchange and sharing of powders between the fish powder group and the sorghum powder group and to prevent mothers from having a preference of what product they wanted for their child after randomisation. After written parental consent was given, the infant was enrolled in the study. Infants received a three-digit participant number allocating them to one of the respective groups.

### 2.6. Nutrient Content of the Fish and Sorghum Powder

Infants in the intervention group received 12 g of dried fish (*chisense*) powder per day, while infants in the control group received 7 g of sorghum powder per day to provide the same energy intake Table 2. The recommended daily allowance for protein for infants 6–12 months of age is 11 g per day [25]. The 12 g of fish powder provided 7.6 g of protein per day to infants in the fish powder group, while 7 g of sorghum powder provided 0.9 g of proteins to the sorghum powder group. Mothers in both groups were given a guideline/example of adding fish/sorghum powder to the infant’s food once per day for six months (either mixing it with cooked food or adding it to food during cooking). Table 2. shows details of the nutrient content of the daily dose of fish or sorghum powders and their contribution to the recommended daily allowances (RDA).

### 2.7. Pilot Study

A pilot study was conducted to test the research instruments and study procedures. Mother–infant pairs (10% of the study sample) attending Shikamushile RHC were recruited. Mothers in the pilot study were asked to make suggestions on the best messages to impart to the participating mothers on information regarding giving the product to the infants (e.g., Traditional sayings). Mothers suggested that the participants should use the following phase “*Abana ukukula bwino, kubapela ubunga bwesabi*” which means “for children to grow well, give them fish powder”. This message was circulated in the villages, and all mothers who were enrolled in the study continued using this phrase to encourage each other. Adjustments were made for all instruments that had errors. The data that were collected from the pilot study was not used for the main study.

Prior to the pilot study, field workers (research assistants and health workers) were trained. The PI facilitated the training. The research assistant’s training included procedures on fish and sorghum powder distribution and how to conduct anthropometric measurements (refer to the methods section below on the standard procedures used). In addition, training on how to record field data was performed. The doctor also trained the nurses in the study on how to conduct an SPT and how to treat infants who may experience fish allergies with antihistamines in accordance.

### 2.8. Measurements

Collection of socio-demographic data, anthropometric measurements, and 24 h dietary recall took place at RHC. A private room was allocated to the study so that there was no interference with the RHC health care routines. Mothers were assigned a time and day in a month when to bring the infant for measurements to avoid congestion and keep mothers waiting for a long time. Data in relation to adherence and illnesses were collected from homes during weekly home visits.

#### 2.8.1. Socio-Demographic Information

A baseline interviewer-administered questionnaire was used to collect socio-demographic information on the mother and their infants aged 6–7 months for both the intervention and control groups. The data collected included age of parents, level of education, occupation, family size, source of income, and marital status. Data on the number of births, breastfeeding, and complementary food practices were also collected.

#### 2.8.2. Anthropometry

Anthropometric measurements were taken using standard procedures [29,30]. The weight, length, and head circumference of infants was taken at baseline and once a month (for six months). For weight measurements, an electronic 2 in 1 SECA (SECA 874, Hamburg, Germany) scale was used. The instrument was calibrated before measurements were taken. Infants were weighed with minimal or light clothing. An average of two measurements were taken. If the second measurement differs from the first measurement by 0.5, then a third measurement was taken. The measurements were recorded to the nearest 0.1 kg.

Length measurements were conducted with a portable UNICEF wooden child measuring length board. The infant had the shoes, hat and any heavy outer clothing removed before taking the measurements. An average of two measurements were taken. If the second measurement differed from the first measurement by 0.5, then a third measurement was taken. The measurements were recorded to the nearest 0.1 cm.

All anthropometric measurements of infant were entered into WHO anthro Version 3.2.2. Software. Weight and length were used to compute z-scores for weight-for-age (WAZ) and length-for-age (LAZ) and were classified according to WHO thresholds.

#### 2.8.3. Dietary Data

A 24-h dietary recall was administered at baseline, follow-up month 2, follow-up month 4, and dietary follow-up month 6. The research assistant recorded the time, type of food that was consumed by the infants in the study, how it was prepared, and how much was consumed. The information was later used to determine the percentage of animal source proteins consumed by infants in the study. Furthermore, it was also used to assess nutrient intake in both groups to further determine food consumption among infants in the study population to be published later.

#### 2.8.4. Skin Prick Test (SPT)

A skin prick test (SPT) was conducted at baseline on all infants in the study to test for fish allergy because even the sorghum powder had a small quantity of fish added in order have a similar fish aroma as the fish product. Infants with positive results were excluded from the study.

##### Procedure

The doctor and nurses performed SPT to assess allergic reaction to fish on all infants aged 6–7 months in the study at baseline. The standard method for testing fish allergies was used [31,32]. The results of the SPT were recorded on the participant data collection form.

#### 2.8.5. Adherence to the Intervention

Seven sachets were parked in small storage bowls to last a week, and infants were given a sachet per day. Research assistants distributed the sachets weekly. Adherence to the intervention was assessed by reports from mothers of infants as well as empty fish/sorghum powder storage bowls each week; the research assistants visited infant’s homes to distribute the fish/sorghum. The number of times that the fish/sorghum powder was consumed during that week and how much was consumed were recorded on the weekly compliance forms. Reasons for non-consumption of fish or sorghum were also recorded. All infants whose mothers did not comply with the intervention for more than two (2) consecutive weeks exited the study.

Ethical approval for the study was obtained from Stellenbosch Human Research Ethics Committee (HREC: M18/10/037), Tropical Disease Research Centre (TDRC: STC 2019/03), Ndola, Zambia, and the National Health Research Authority Lusaka, Zambia. Permission to conduct a study at Shikamushile RHC was also obtained from Samfya District Health Office (SDHO). Written informed consent was obtained from the mothers to allow their children to participate in the study.

### 2.9. Statistical Analysis

Completed questionnaires were checked for completeness, coded, and entered into Microsoft Excel (version 2016). The data were then checked for missing values and exported to STATA (version 16) for analysis. Descriptive statistics were used to compute means, standard deviations (SDs), frequencies, and percentages. Confounding factors known to adversely affect growth were investigated. An independent *t*-test was used to assess the mean difference between the fish powder group and sorghum powder group, and no statistically significant differences were observed between the two groups (baseline stunting *p* = 0.091 and animal source food consumption before addition of fish powder *p* = 0.0924). Additionally, none of baseline socio-economic characteristics were associated with growth among infants *p* > 0.05. Nevertheless, malaria cases showed a significant difference in growth among infants in the sorghum powder group (*p* = 0.001).

A linear mixed-effects model was used to do an intention to treat analysis for the primary outcome LAZ. The model included an indicator variable for the intervention, a discrete-time variable, and the intervention and time variable interaction. The test for a significant interaction effect between intervention and time was the overall test for an intervention effect. The estimation for the model parameters was performed via full maximum likelihood. This was the imputation approach used to handle the missing data at the time points post-randomisation to facilitate the intention to treat analysis. The estimated intervention effects at each month and 12 months considered the baseline difference at randomisation and used the difference-in-difference approach to estimate the mean difference as the intervention effect as well as the 95% confidence interval. The baseline effect was thus excluded from the intervention effect (this was the same as starting from zero difference at baseline).

A *p*-value of *p* < 0.05 represented statistical significance, and 95% confidence intervals were used to describe the precision of the estimation of the unknown parameters. All statistical analyses were performed using STATA software version 16.

## 3. Results

Two hundred eighty-seven (287) 6–7-month-old infants were screened for eligibility. Of these screened, 49 infants were excluded as they did not meet the study criteria. After randomisation, three infants had insufficient data at baseline. The final study population consisted of 235 participants at baseline (intervention: 118 and control: 117). Of the 235, 16 withdrew from the study, while 33 did not show up at follow-up. Therefore, the total number of participants who completed the 6 months intervention was 186 (100 intervention: 100 and control: 86), Figure 1.

### 3.1. Baseline Characteristics of the Study Population

#### 3.1.1. Baseline Characteristics of Participating Infants and Their Mothers by Group

The baseline characteristics of the infants included in the study are depicted in Table 3. Of the 235 infants included, 125 (53.4%) were male, while 110 (46.7%) were female. The baseline characteristics were similar for both the fish and sorghum groups. The mean age of infants was 6.64 (±0.54) and 6.63 (±0.50) months for the fish powder and sorghum powder groups, respectively. It was found that in both groups, all infants (100%) were still breastfeeding; most infants were introduced to solid food at five months. The prevalence of stunting for infants at baseline was very high (78.3%), with 74.6% (*n* = 88) in the fish group and 82.1% (*n* = 96) in the sorghum group being stunted. These baseline stunting measurements between the fish powder group and sorghum powder group were not significantly different (*p* = 0.091).

The anthropometric and socio-demographic information of the infant’s mothers is shown in Table 3. The percentage of married mothers in the fish powder group was 93% (*n* = 110), while 97% (*n* = 113) were from the sorghum powder group. Most of the mothers in both the fish (*n* = 64; 54.2%) and sorghum (*n* = 85; 73.3%) powder group had attained primary education and very few in both the fish (*n* = 18; 15.0%) and sorghum (*n* = 12; 10.4%) groups had no formal education. The number of births experienced ranged from one to eight births, and the family size ranged from three to ten people per household.

The overall prevalence of stunting among mothers in the study was 60.3% (*n* = 141), and the mean body mass index BMI was 22.6 kg/m^2^ ± 1.48, ranging from 17.9 kg/m^2^ to 26.9 kg/m^2^. Among the stunted, 71 (60.2%) were from the fish powder group, while in the sorghum group, 70 (60.3%) mothers were stunted. A similar BMI was found in the fish powder group [22.59 kg/m^2^ ± 1.48 (18.1–26.7 kg/m^2^)] and in the sorghum group [22.51 kg/m^2^ ± 1.48 (17.9–26.9 kg/m^2^)].

#### 3.1.2. Socio-Economic Characteristics of Mothers of Infants in the Study

Table 4 represents the socio-economic characteristics of mothers of infants in the study. Most mothers were housewives (*n* = 221; 94.4%) with almost equal numbers in the fish and sorghum groups (94.9% and 93.9%, respectively). The monthly household income was low, K500 ($50), and similar in the two groups. The main source of fuel used was firewood, with equal destitution between the groups [106 (89.8%) in the fish group and 107 (92.2%) in the sorghum group]. Very few participants in the study owned domestic animals or had backyard vegetable gardens. More than half of the population had not owned domestic animals (fish powder group 57.6%, sorghum powder group 50%). The main source of drinking water was shallow wells (*n* = 170; 72.3%), and all participants had a pit latrine. Additionally, none of the socio-economic characteristics listed in Table 3 were associated with stunting *p* > 0.05.

### 3.2. The Effect of the Fish Powder on the Growth of Infants

#### Infants Growth for the Study Duration and the Effect of the Intervention

The effect of the fish powder on the infants’ growth between the fish and sorghum groups as assessed by linear mixed-effect analysis is presented in Table 5. There was a significant difference in the mean LAZ between the fish powder and sorghum powder groups at the baseline and endpoint of the intervention. Infants in the fish powder group had improved linear growth compared to the sorghum group. The effect of fish powder on LAZ was 1.26 (95% CI: 0.94–1.57) at the endpoint. Furthermore, a significant intervention effect was found between the fish and sorghum groups for WAZ (*p* < 0.05).

Fish powder additionally reduced the levels of stunting in the fish powder group by 25.6% (from 74.6–49%).

#### Length for-Age z-Scores

The effect of the intervention over time is presented in Table 6. The results show that fish powder improved the linear growth of infants in the fish powder group. The mean LAZ (from the first month (1) of follow-up to the last month (6) of follow-up) of the fish powder and sorghum powder groups differed over time. There was a significant difference in the mean LAZ in the fish powder group compared to the sorghum powder group over all the months (*p* = 0.0001).

**Figure 2:**
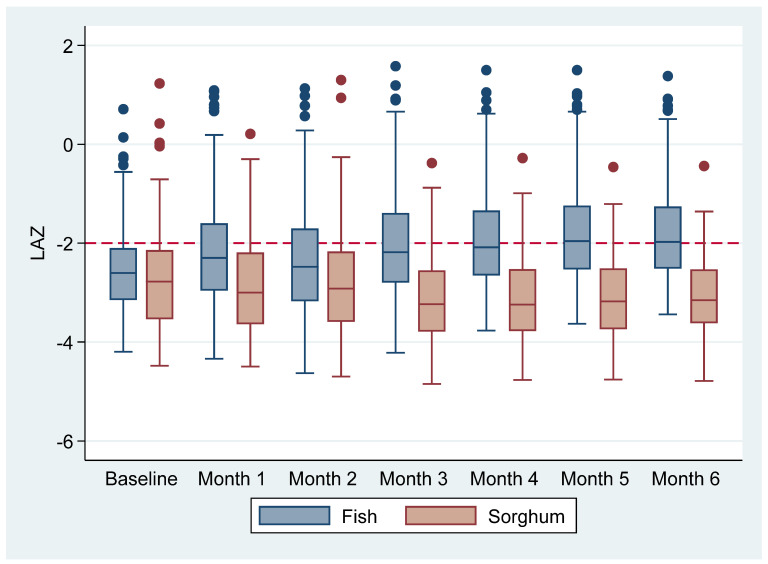
Boxplots of changes in length-for-age z-score (LAZ) over time between the fish powder group and sorghum powder group.

Figure 2 shows the effect of fish powder on LAZ compared to sorghum powder in boxplots over time by the group. The infants in the fish powder group had improved LAZ at each month of follow-up (1–6) compared to infants in the sorghum powder group. It can be observed that in the second month of follow-up, the infants in the sorghum had a slight increase in LAZ, whereas the LAZ decreased in the following months. The results further showed that the most significant effect in the fish powder group was earlier in the intervention (first three months), while no significant change was observed in the latter months (months 4–6) of the study (*p* = 0.2915).

Sensitivity analysis of the intervention effect of fish powder on LAZ at each month of follow-up, excluding the first group of enrolled infants whose baseline measurements were taken at seven months, is shown in Table 7. There was a reduction in the mean intervention effects between the fish powder and sorghum powder at the endpoint from 1.26 to 1.09. The sensitivity analysis effect size is less than what was observed in the fourth month of follow-up before excluding the first group of infants. Additionally, there was no significant difference in the LAZ effect (*p* > 0.05) in the second month of follow-up between the two groups. This was not the case in the analysis that included the first group of infants, where all follow-up months had a significant difference. These results show that the first group of infants enrolled at seven months had contributed to the high effect size observed in the study because of the one-month advantage into the intervention (fish powder consumption) compared to the rest of the infants.

### 3.3. Morbidity Reported in the Infants during the Six-Month Intervention Period

The types of illness experienced by infants in the study during the six-month intervention period are presented in Table 8. The largest percentage did not report any illness, which was 182 infants (77.4%). The number of infants who had experienced at least one form of illness in the study was 53 (22.6%). For almost all infants for whom illness was reported during the study, diarrhoea was experienced at some point (51 of the 53 infants). The incidence of diarrhoea was higher in the sorghum group than in the fish group (fish: 22, sorghum: 29), while vomiting was the same in both groups (12 incidences). Two infants in the sorghum group experienced a skin rash, while none in the fish powder group had reported skin rashes. The occurrence of coughing and malaria episodes was also higher in the sorghum powder group compared to the fish powder group. Malaria cases in the sorghum powder group had a negative effect on the growth of infants in the group (*p* = 0.001).

### 3.4. Dietary Data

None of the infants were reported to have consumed fish at baseline in both groups (Table 9). The intervention, as expected, increased the intake of fish in the intervention group to 100% from dietary follow-up months two, four, and six. The sorghum group had less consumption throughout the study, with 8.2%, 3%, and 10.5% recorded at two, six, and eight dietary follow-up months, respectively. Consumption of meat and poultry was low in both groups. The fish group’s highest meat and poultry consumption was 6%, while the sorghum group was 3.5%, with both groups recording less than that in both groups. Dairy products were extremely low, with both groups recording 0.9% as the highest consumption throughout the follow-up months. The daily consumption of animal source proteins in the sorghum powder group and the fish powder before the addition of fish was not significantly different (*p* = 0.0924).

## 4. Discussion

Fish protein offers the same protein quality as other animal source proteins such as meat, eggs, chicken, or liver. A cluster randomised controlled trial that assessed the effect of a fish protein isolate (FPi), administered over six months, on the growth of children aged 6–36 months compared with a standard meal (with animal protein derived from beef, chicken, eggs or liver) found that fish protein was just as effective in improving growth (HAZ) of children as a standard meal [35]. Fish consumption has shown an improvement in linear growth among children in other studies as well [36,37]. Observation of fish consumption and non-fish consumption in a cross-sectional three-month survey that was conducted in Sungai Pinang, Banjar District, Indonesia, among children aged 6–24 months, showed that children who were not consuming fish were 5.5 times (80.9%) more likely to be stunted compared to those who consumed fish (19.1%) [36] The effect on linear growth after fish consumption was also observed in a South Malawi study that assessed consumption of animal source proteins (ASP) and its association with improved HAZ in rural children aged 12–36 months. Results showed that HAZ increased by 0.3 cm among children aged 12–36 months who had consumed fish as the main ASP [37].

The present study found that fish powder improved linear growth (LAZ) among infants in the Samfya District by 1.26 (95% CI, 0.94–1.57) over the six-month study period. Further sensitivity analysis (removing the first group of infants that had their baseline measurements at seven months, refer to Figure 1) showed a reduction in the LAZ effect size between the fish powder and the sorghum powder from 1.26 (95% CI, 0.94–1.57) to 1.09 (0.71–1.47). This effect size was still larger than reported in other studies that accessed ASP complementary foods in low-income communities [38,39].

There was only one study in Lima, Peru, that assessed the efficacy of fish in the early complementary feeding to improve growth in infants and young children in low-income countries to which we could compare our results [35]. However, the Peru study compared the FPi to a standard diet with animal protein derived from beef, chicken, eggs, or liver among children aged 6–36 months. There was no notable difference in growth between children who consumed FPi and those who consumed the standard protein diet [35]. Fish was found to be a potential animal-source protein to improve growth just such as other ASP (beef, chicken, eggs, or liver) and cheaper than other animal source proteins [35].

Nevertheless, other complementary feeding trials (in Ecuador and Malawi) used other animal protein sources such as eggs in the early complementary feeding to improve children’s growth compared to the usual dietary intake [38,39]. In Ecuador, an egg per day for six months of intervention had a significant effect on growth with a mean LAZ effect of 0.63 (95%CL, 38–0.88) [38]. The current study used repeated measurements, which showed the change of LAZ over time, and all the dropouts were incorporated into the analysis (refer to Section 2.8 on statistical analysis), unlike the Ecuador study, which only included the baseline and endpoint HAZ measurements, without knowing what happened in the follow-up months between baseline and endpoint.

The Malawi study did not find any significant effect of egg consumption on child growth 0.07 (95%CL, −0.01–0.14). The low effect was associated with the low prevalence of stunting (14%) and high fish consumption (60%) in their usual dietary intake apart from eggs at six months follow-up, giving the control groups access to other ASP foods [39]. The prevalence mean LAZ at baseline was also very low (−0.9). This was not the case in the current study, which had the prevalence of stunting at 78.3%, and fish as the main ASP that was available in the Samfya District, where the usual dietary intake among infants was predominantly plant-based (green leaf vegetables), with less than 7% of infants consuming other ASP in both groups (refer to Table 9). The mean LAZ of −2.6 at baseline in the current study was lower than the study in Malawi at −0.9. Additionally, the International Livestock Research Institute (ILRI) reported that children with higher levels of stunting benefit more from the ASP food interventions than the healthy or less stunted [40]. Secondary data of two RCTs were used to assess the type and protein quality in association with stunting, environmental enteric dysfunction (EED), and acute malnutrition in southern Malawi by Kaimila et al. and found that fish consumption improved height-for-age among children aged 12–36 months [37].

The high levels of stunting in our study could be the reason for the larger effect sizes observed in the current study compared to the Malawi and other studies. Sorghum is known to contain tannin, which could affect the bioavailability of iron absorption in the body, which may negatively affect linear growth in children. The tannin levels could thus have affected iron bioavailability, thus contributing to the low LAZ observed in the sorghum group [41]. However, only 7 g of sorghum powder was given to the infants per day (not a full bowl), thus making its presence negligible.

Furthermore, within the fish powder group of the current study, no difference in the LAZ effect was observed in the last three months of follow-up (*p* = 0.2915), as was seen from the first month of follow-up to the third month of the intervention. Even though a trend in growth was observed in the last three months by 0.12 (from 1.14–1.26), it was not statistically significant. However, the LAZ effect of 0.63 (from 0.63–1.26) between follow-up month one and follow-up month six was significant (*p* = 0.0001). The first three months might have shown rapid growth because this period coincided with the harvest season when locally grown crops were in abundance, while the last three months were during the planting season when food is known to be scarce. The abundance of food meant that infants might have been given more food with fish/sorghum powder added in the first three months compared to the last three months.

The current study further found that ASP from fish powder reduced levels of stunting among infants in the fish group by 25.6.2% (from 74.6% at baseline to 49.0% at endpoint). These findings are similar to the Ecuador egg study by Iannott et al., which also recorded a reduction in the levels of stunting by 47% (prevalence ratio [PR], 0.53; 95% CI, 0.37–0.77) [38]. Additionally, consumption of ASP was found to reduce stunting in other studies that assessed animal source food (ASF) consumption. In Nepal, a study by Miller et al. reported that stunting was reduced by 16% among children aged 6–24 months [42]. Animal source protein from milk was also associated with reduced stunting among children aged 6–59 months in a systematic review that assessed milk consumption and its association with growth in children from low- and medium-income countries (1.9%; 95% CI, −0.02, −0.01) [43].

Morbidities reported during the six months of the study included diarrhoea, coughing, vomiting, skin rash, and malaria. A bigger percentage (77.4%) of infants did not experience any illness. However, among those that experienced an illness (22.6), diarrhoea was the most prevalent illness in both groups, with the control group having more cases than the intervention group. Additionally, none of these illnesses were due to fish powder consumption, as the skin prick test conducted on all infants who presented illnesses was negative. Furthermore, none of the children experienced a skin rash (suggestive of a possible related allergy) during this period [44]. However, further analysis showed that malaria was negatively associated with growth in the sorghum powder group (*p* = 0.001). Infants who had malaria in the sorghum powder group were less likely to have improved linear growth than those who had no malaria. This might have been a random outcome because there were very few malaria cases in the study. Nevertheless, disease burden such as malaria has been associated with poor growth and stunting among children below five [45]. Therefore, the malaria incidence in the sorghum powder group may be causation or con-sequential to the study results.

The food consumption recorded from all the 24-h dietary recalls (at baseline, showed low consumption of animal source foods in both the fish and sorghum groups. However, after adding the fish powder to the complementary foods, the ASP intake. As expected, it increased to 100% in the fish powder group in all the three-dietary follow-ups months compared to the sorghum powder group. ASP from fish is highly bioavailable and offers the same protein quality as meat, eggs, chicken, and liver, and thus a good protein source for infants [35]. A study by Grillenberger et al. showed that plant source nutrients, including protein from plant-based sources, are associated with poorer growth than animal source nutrients [46]. Thus, animal-sourced protein from fish powder contributed to growth among infants in the fish powder group.

A positive relationship between fish powder consumption and improved linear growth among infants was seen in this study. Fish improved linear growth among infants in the Samfya District. These results show that fish can potentially improve growth among children in places such as Samfya, where it is likely an underutilised protein source despite its availability.

As observed at baseline and during follow-up months in the sorghum powder group, mothers in the Samfya district did not give fish to their children. According to studies, some of the reasons mothers do not feed fish to children include cultural beliefs, lack of education, and lack of knowledge on the importance of protein from fish to infants. Cultural beliefs and lack of education were reported to have been associated with low consumption of fish in a study conducted in India that looked at cultural factors and their correlation with the occurrence of stunting among children aged 6–24 months [36]. One of the beliefs established in the current study was that mothers in the Samfya district do not provide small fish to their infants due to the fear of choking. Even though the district has larger fish species that can be given to infants, it is mostly sold to other parts of the country to generate income, thus making it more expensive to the poor households compared to the small fish used in the current study.

## 5. Conclusions

The current study’s findings agree with previous studies that consumption of ASP in the early complementary feeding period improves linear growth and helps in the reduction of stunting among children below five years [19,37,47,48]. Thus, ASP and fish powder as an available protein source in an area where plant-based complementary feeding is mostly practiced led to the increased linear growth that was observed in the current study. Other confounding factors such as baseline stunting (74% vs. 82%) and animal source food consumption before the addition of fish powder were not significantly different between the two groups. However, malaria cases and tannins in the sorghum powder group could have had an effect on growth. Therefore, fish powders can potentially be used as an early complementary food to improve the growth of infants in low-income communities with low animal-source protein consumption.

## Figures and Tables

**Table 1:** Recruitment schedule.

Enrolment Group	Year/Month of Study									
	2019	2019	2019	2019	2019	2019	2019	2019	2019	2020
	April	May	June	July	Aug	Sep	Oct	Nov	Dec	Jan
		1	2	3	4	5	6	7	8	9
1 (*n* = 78)	(Baseline) *	Baseline						Exit		
2 (*n* = 47)		Baseline						Exit		
3 (*n* = 51)			Baseline						Exit	
4 (*n* = 62)				Baseline						Exit
Total (*n* = 238)										

* Due to inaccurate measurements at the baseline assessment in the first group of participants enrolled, the measurements for this group at the first follow-up were used as the baseline, and the group was followed for an additional month.

**Table 2:** The nutrient content of the daily dose of fish, sorghum and their contribution to the RDA/AI.

Nutrients	RDA/AI for 6–12 Month Infants ^†^	Nutrients per 12 g Fish Powder ^‡^	% of RDA (Fish Powder) ^¥^	Nutrients per 7 g Sorghum Powder	% of RDA (Sorghum Powder)
Energy (kcal)		25	-	25	-
6–12 months					
Protein (g)	11.0	7.6	69.0	0.9	10.0
Fat (g)	30.0	1.1	4.0	0.2	0.6
Carbohydrates (g)	95.0	-	0.0	5.0	5.3
EPA^3^ (g) *	0.5	0.1	20.0	-	-
DHA^3^ (g) **	0.5	0.1	20.0	-	-
Calcium (mg)	260.0	260.0	100.0	1.4	0.5
Iron (mg)	11.0	1.0	9.0	0.3	0.0
Vitamin A (ug)	500.0	34.0	13.0	-	-
Nicotinic acid (mg)	4.0	0.7	17.5	0.2	5.0
Zinc (mg)	3.0	1.6	52.0	0.2	7.0

Source: ^†^ RDA/AI estimates are reported from the Institute of Medicine of the National Academies. Dietary reference intakes: The Essential Guide to Nutrient Requirements (2006) and the Food and Nutrition Board, Institute of Medicine NA. Dietary Reference Intakes (2011) [26,27], ^‡^ Nutrient content of fish as reported by Longley et al. (2014) [23], ^¥^ nutrient content of sorghum powder was taken from the Zambian Food Composition Tables (2009) [28] * Eicosapentaenoic acid (EPA, 20:5 (*n* − 3)) and ** docosahexaenoic acid (DHA, 22:6 (*n* − 3).

**Table 3:** Baseline characteristics of participating infants and their mothers by group.

	Fish Powder (*n* = 118)		Sorghum Powder (*n* = 117)	
Characteristic	*n* (%) or Mean ± SD	Range	*n* (%) or Mean ± SD	Range
**Infants**				
**Gender**				
Male	63 (53.4)	-	62 (53.0)	-
Female	55 (46.6)	-	55 (47.0)	-
**Gestation age (weeks)**	38.4 ± 1.1	37.0–44.0	38.2 ± 1.0	36.0–41.0
**Number of low-birth-weight infants ^§^**	1 (0.9)	-	1 (0.9)	-
**Age** (months)	6.6 ± 0.5	5.9–7.9	6.6 ± 0.5	6.0–7.9
**Infant still being breastfed**	118 (100)	-	117 (100)	-
**Age of solid food introduction (months)**	5.2 ± 0.5	3.06.0	5.1 ± 0.6	1.0–6.0
**Anthropometry**				
Birth weight (kg)	3.2 ± 0.4	2.1–4.6	3.1 ± 0.4	2.4–4.0
Weight (kg)	6.9 ± 0.9	5.7–9.9	6.7 ± 0.7	5.2–9.6
Length (cm)	62.3 ± 2.7	58.6–72	61.5 ± 1.7	57.3–66.5
Head circumference (cm)	41.4 ± 1.8	39–45.1	44.3 ± 34.9	39.3–44.9
Mid upper arm circumference (cm)	13.2 ± 0.6	12.5–14.9	13.1 ± 0.6	11.5–15.5
**Anthropometry classifications**				
Stunting ^†^	88 (74.6)		96 (82.1)	
Underweight ^‡^	23 (19.5)	-	27 (23.1)	-
Wasting ^֏^	0 (0.0)		0 (0.0)	-
Overweight	11 (9.3)	-	9 (7.7)	-
Length-for-age z-score (LAZ)	−2.51 ± 1.12	−4.4–1.2	−2.71 ± 0.87	−4.5–−0.7
Weight-for-age z-score (WAZ)	−1.1 ± 1.0	−2.7–1.9	−1.4 ± 0.9	−3.0–1.2
Weight-for-length z-score (WLZ)	0.7 ± 0.8	−1.0–4.4	0.6 ± 0.9	−1.8–4.4
Head-circumference-for-age z-score (HCZ)	−1.4 ± 1.1	−1.4–1.1	−1.7 ± 1.0	−3.4–1.1
**Mothers**	118.0 (50.4)	-	116.0 (49.6)	-
**Age** (years)	28.71 ± 6.7	18.1–45.8	29.5 ± 6.3	17.4–42.6
Anthropometry				
Weight (kg)	49.9 ± 3.6	40.5–61.2	49.7 ± 3.6	39.5–63.0
Height (m)	1.5 ± 0.1	1.4–1.6	1.5 ± 0.0	1.4–1.7
BMI (kg/m^2^) ^¥^	22.6 ± 1.5	18.1–26.7	22.5 ± 1.5	17.9–26.9
**Maternal stunting (%) ^†^**	71 (60.2)	-	70 (60.3)	-
**Number of births**	3.3 ± 1.9	1.0–8.0	3.6 ± 1.8	1.0–8.0
**Family size**	5.4 ± 1.9	3.0–10.0	5.6 ± 1.9	3.0–10.0
**Marital status**				
Single	5.0 (4.0)	-	3.0 (2.6)	-
Married	110.0 (93.0)	-	113.0 (97.4)	-
Divorced	3.0 (3.0)	-	0.0 (0)	-
**Education level**		-		-
No formal education	18.0 (15.3)	-	12.0 (10.4)	-
Primary level	64.0 (54.2)	-	85.0 (73.2)	-
Secondary level	36.0 (30.5)	-	19.0 (16.4)	-

^§^ Babies who are born weighing less than 2500 g. ^†^ Stunting is length-for-age < −2 SD below the WHO Child Growth Standards median, underweight ^‡^ is weight-for-age < −2 SD below the WHO Child Growth Standards median wasting ^֏^ is weight-for-Length < −2 SD below the WHO Child Growth Standards median and overweight is > +2 SD above the WHO Standard median [4] ^¥^ BMI < 18.5 kg/m^2^ indicates underweight, BMI 18.5–24.9 kg/m^2^ indicates normal weight, BMI 25.0–29.9 kg/m^2^ indicates overweight, BMI ≥ 30.0 indicates obesity. ^†^ Stunting in mothers was defined as a height less than 1.5 m [33,34]. ^‡^ One of the mothers in the sorghum powder group was a legal guardian; thus, no information was collected.

**Table 4:** Socio-economic characteristics of participating mothers of infants in the study by group.

	Fish Powder (*n* = 118)	Sorghum Powder (*n* = 116) ^‡^
Characteristic	*n* (%)	*n* (%)
**Occupation**		
Housewife	112.0 (94.9)	109.0 (93.9)
Farmer	0.0 (0.0)	3.0 (2.6)
Own business	2.0 (1.7)	1.0 (0.9)
Formal employment	1.0 (0.8)	0
Stays with parents	3.0 (2.5)	3.0 (2.6)
**Source of income**		
Salaried job	0.0 (0)	3.0 (2.6)
Husband	109.0 (92.4)	108.0 (93.1)
Own business	5.0 (4.2)	2.0 (1.7)
Parents	4.0 (3.4)	3.0 (2.3)
**Monthly Income**		
Below K500 ($50)	117.0 (99.2)	114.0 (98.3)
K500–K99 ($50–$99)	1.0 (0.8)	2.0 (1.7)
**Type of fuel**		
Electricity	0.0 (0)	1.0 (0.9)
Solar	11.0 (9.0)	8.0 (6.9)
Charcoal	1.0 (1.0)	0.0 (0.0)
Firewood	106.0 (90.0)	107.0 (92.2)
**Domestic animal ownership**		
Chickens	38.0 (32.2)	46 (39.7)
Goats	10.0 (8.5)	10 (8.6)
Pigs	2.0 (1.7)	2 (1.7)
None	68.0 (57.6)	58 (50.0)
**Garden ownership**		
Green leafy vegetables	4.0 (3.4)	3 (2.6)
Red vegetables(e.g., tomatoes)	1.0 (0.9)	0 (0.0)
None	113.0 (95.7)	113 (97.4)
**Source of drinking water**		
Mono pump	34.0 (28.8)	29.0 (25.0)
Shallow well	83.0 (70.3)	86.0 (74.1)
Lake Bangweulu	1.0 (0.9)	1.0 (0.9)
**Type of toilet**		
Pit latrine	118.0 (100.0)	117.0 (100.0)

^‡^ One of the mothers in the sorghum powder group was a legal guardian; thus, only in the *n* = 116 in the sorghum powder group.

**Table 5:** Infants growth for the study duration and the effect of the intervention.

	Fish Powder		Sorghum Powder		Intervention Effect ^‡^	
	Baseline (*n* = 118)	End Point (*n* = 100)	Baseline (*n* = 117)	End Point (*n* = 86)		
	Mean ± SD	Mean ± SD	Mean ± SD	Mean ± SD	Mean Difference^2^ (95% CI)	*p*-Value
	n (%)	n (%)	n (%)	n (%)		
LAZ ^†^	−2.51 ± 1.12	−1.76 ± 1.09	−2.71 ± 0.87	−3.12 ± 0.84	1.26 (0.94–1.57)	<0.001
WAZ	−1.09 ± 1.06	0.10 ± 0.66	−1.25 ± 0.89	−0.95 ± 0.68	0.95 (0.6–1.23)	<0.001
Stunting	88 (74.6)	49 (49)	96 (82.1)	78 (90.1)		

^†^ LAZ (length-for-age z-scores), WAZ (weight-for-age z-scores), ^‡^ Mean difference is the difference in the mean LAZ, WAZ, between the fish and sorghum powder group at the endpoint of the six-month intervention period.

**Table 6:** The intervention effect of the fish powder on LAZ at each month of follow-up.

	Fish Powder		Sorghum Powder		Intervention Effect ^†^	
Month of Follow-Up	*n*	LAZ Mean ± SD	*n*	LAZ Mean ± SD	Mean Difference ^‡^ 95% CI	*p*-Value
Follow-up month 1	115	−2.13 ± 1.18	111	−2.93 ± 0.93	0.63 (0.40–0.87)	<0.001
Follow-up month 2	112	−2.35 ± 1.17	110	−2.79 ± 1.13	0.26 (0.02–0.50)	0.032
Follow-up month 3	110	−1.99 ± 1.16	104	−3.16 ± 0.90	1.02 (0.77–1.28)	<0.001
Follow-up month 4	108	−1.88 ± 1.11	100	−3.15 ±.0.87	1.14 (0.87–1.41)	<0.001
Follow-up month 5	103	−1.78 ± 1.11	90	−3.13 ± 0.85	1.24 (0.95–1.53)	<0.001
Follow-up month 6	100	−1.76 ± 1.09	86	−3.12 ± 0.84	1.26 (0.94–1.58)	<0.001

^†^ Intervention effect as estimated by the linear mixed model. ^‡^ The mean difference is the difference between the mean LAZ in the fish powder group and the sorghum powder group at each month of follow-up.

**Table 7:** The intervention effect of the fish powder on LAZ at each month of follow-up as assessed using liner mixed effect sensitivity analysis.

	Fish Powder		Sorghum Powder		Intervention Effect ^†^	
Month of Follow-Up	*n*	LAZ Mean ± SD	*n*	LAZ Mean ± SD	Mean Difference ^‡^ 95% CL	*p*-Value
Follow-up month 1	78	−2.37 ± 0.96	73	−2.84 ± 0.92	0.47 (0.19–0.76)	<0.001
Follow-up month 2	75	−2.56 ± 1.06	72	−2.72 ± 1.19	0.16 (−0.12–0.46)	0.268
Follow-up month 3	74	−2.22 ± 0.93	66	−3.02 ± 0.87	0.84 (0.53–1.16)	<0.001
Follow-up month 4	72	−2.12 ± 0.87	64	−3.05 ± 0.88	0.98 (0.65–1.31)	<0.001
Follow-up month 5	69	−2.06 ± 0.85	58	−3.04 ± 0.84	1.07 (0.72–1.42)	<0.001
Follow-up month 6	66	−2.04 ± 0.83	57	−3.06 ± 0.84	1.09 (0.71–1.47)	<0.001

^†^ Intervention effect as estimated by the linear mixed model sensitivity analysis. ^‡^ The mean difference is the difference between the mean LAZ in fish powder group and the sorghum powder group at each month of follow-up.

**Table 8:** Morbidities reported in the infants during the six-month intervention period.

	Fish Powder (*n* = 118)	Sorghum Powder (*n* = 117)	Total (*n* = 235)
	*n* (%)	*n* (%)	*n* (%)
Total number of illnesses			53 (22.6)
Type of illnesses ^†^			
Diarrhoea	22 (18.6)	29 (25.6)	51 (21.7)
Vomiting	12 (10.2)	12 (10.3)	24 (10.2)
Coughing	8 (6.7)	16 (13.7)	24(10.2)
Malaria	3 (2.5)	15 (12.8)	18 (7.7)
Skin rash	0 (0)	2 (1.7)	2 (0.9)
Total number of illnesses	25 (21.2)	28 (23.9)	53 (22.6)
Had no illness	93 (78.8)	89 (76.1)	182 (77.4)

^†^ Type of illnesses: experienced by infants in the study during the six-month intervention period

**Table 9:** Animal source food consumption by intervention group.

	Fish Powder Group	Sorghum Powder Group
Variable	*n* (%)	*n* (%)
Baseline	118	117 (100)
Consumption of fish	0 (0.0)	0 (0.0)
Consumption of meat and poultry	1 (0.8)	1 (0.9)
Consumption of dairy	1 (0.8)	1 (0.9)
Dietary follow-up month 2	112	110
Consumption of fish	112 (100)	9 (8.2)
Consumption of meat and poultry	3 (2.7)	1 (0.9)
Consumption of dairy	1 (0.9)	0 (0.0)
Dietary follow-up month 4	108	100
Consumption of fish	108 (100)	3 (3.0)
Consumption of meat and poultry	5 (4.6)	2 (2.0)
Consumption of dairy	0	0
Dietary follow-up month 6	100	86
Consumption of fish	100 (100)	9 (10.5)
Consumption of meat and poultry	6 (6.0)	3 (3.5)
Consumption of dairy	0 (0.0)	0 (0.0)

## Data Availability

The data presented in this study are available on request from the corresponding author. The data are not publicly available due to ethical restrictions.
